# Knowledge of Physiotherapy Practice among Medical Interns in a Tertiary Care Hospital: A Descriptive Cross-sectional Study

**DOI:** 10.31729/jnma.6887

**Published:** 2021-08-31

**Authors:** Prakash Kumar Mahto, Naresh Manadhar, Sunil Kumar Joshi

**Affiliations:** 1 Department of Physiotherapy, Kathmandu Medical College, Kathmandu, Nepal; 2 Department of Community Medicine, Kathmandu Medical College, Kathmandu, Nepal

**Keywords:** *internship*, *knowledge*, *medical*, *modalities, physical therapy, practice*

## Abstract

**Introduction:**

Medical doctors have profound influence on other health professions including physiotherapist as they are at the top of the pyramid of healthcare profession. There is a lack of knowledge of physiotherapy among medical doctors. They may not be knowing of all physiotherapy services and practice. The objective of this study was to find adequate knowledge of physiotherapy practice among medical interns in a tertiary care hospital.

**Methods:**

A descriptive cross-sectional study was conducted on medical interns of a tertiary hospital of Kathmandu between 21^st^ March - 20^th^ May 2021, after taking ethical approval from the Institutional Review Committee. A convenient sampling method was used and sample size was calculated to be 94. A structured questionnaire was used to collect the demographic details and knowledge of Physiotherapy. Data was analyzed using statistical package for the social sciences version 20. Point estimate at 95% Confidence Interval was calculated along with frequency and percentage for binary data.

**Results:**

The knowledge of physiotherapy practice was seen adequate among 16 (17%) (95% Confidence Interval = 9.41-24.59) medical interns. Physiotherapy is effective in reducing pain was acknowledged by 89 (97.4 %), 61 (64.9 %) had knowledge about conditions treated by physiotherapy, 55 (58.5 %) had knowledge that physiotherapy treatment follows definite treatment protocol and 26 (27.7 %) had knowledge that exercise prescription is done in physiotherapy.

**Conclusions:**

The prevalence of adequate knowledge is less in our study which is similar to other studies done in similar settings. Therefore, there is a need of educating the future medical doctors about physiotherapy, thereby reaching a better patient care.

## INTRODUCTION

There has been a tremendous change over the attitude of the medical community regarding physiotherapy. The sole people considered appropriate to take care of any injured person were a medical doctor. Today, health practitioners work alongside physiotherapists to offer the best treatment and recovery choices for their patients.^1^ Medical doctors have profound influence on other health professions including physiotherapist as they are at the top of the pyramid of healthcare professionals.^2^ As a result, patients still rely on the doctor for recommendations to other healthcare professionals although physiotherapy is now universally acknowledged as an autonomous profession.^3^

Despite the recognition and advanced gain worldwide in physiotherapy, there is a lack of awareness of physiotherapy among medical doctors.^4^ Medical professional may not have an adequate knowledge of all physiotherapy services.^5,6^

The objective of this study was to find adequate knowledge of physiotherapy practice among medical interns in a tertiary care hospital.

## METHODS

A descriptive cross-sectional study was conducted on medical interns of a tertiary hospital of Kathmandu between 21^st^ March - 20^th^ May 2021. Informed consent was obtained from all the participants and information collected kept confidential. Ethical approval was taken from the Institutional Review Committee (Ref no. 2603202106). The inclusion criterion was medical interns who had joined their internship after passing their final Bachelor of Medicine and Bachelor of Surgery (MBBS). The exclusion criteria being interns who were infected by COVID 19 and those who were not physically present.

Sample size was calculated by the formula

n = Z^2^ × p × q / e^2^

  = (1.96)^2^ × 0.5 × 0.5 / (0.11)^2^

  = 80

where,

n = sample size
Z = 1.96 at 95% confidence interval
p = prevalence of adequate knowledge of physiothrapy for maximum sample size, 50%
q = 1-p = 0.5
e = margin of error, 11%

However, the total sample size taken was 94. Sampling method used was convenient sampling. The data was collected by using a structured questionnaire developed with reference to WHO standard questionnaire for Knowledge. The questionnaire included two parts. The first part included demographic information of the participants and the second part included opinion and knowledge about physiotherapy. The pre-testing of the questionnaire was done in 10 % of the sample i.e., ten medical interns out of actual study participants, sampled conveniently. Those pre-tested medical interns were not included for the main study. Then, the questionnaires, its content, wording, instructions and ease of completion were revised accordingly, and final questionnaire was drafted. Responses were recorded on a three-point Likert scale, in terms of "YES", "NO" or "Don't Know". The knowledge was estimated using proportion. Knowledge about physiotherapy was determined according to the scoring awarded to the population as adequate & inadequate knowledge. The collected data was analysed using statistical package for social sciences (SPSS) version 20.

## RESULTS

The knowledge of physiotherapy practice was seen adequate in 16 (17 %) (95% Confidence Interval= 9.41-24.59) interns (Table 1).

**Table 1:** Knowledge of Physiotherapy practice among medical interns.

Knowledge	n (%)
Adequate	16 (17)
Inadequate	78 (83)
Total	94 (100)

**Figure 1:**
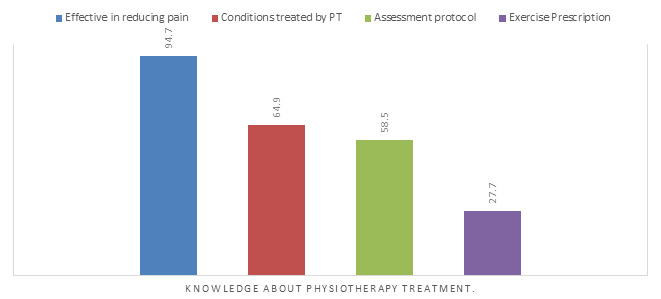
Knowledge about physiotherapy treatment.

Physiotherapy is effective in reducing pain was acknowledged by 89 (94.7%) medical interns (Figure 1). Eighty-three (88%) respondents agreed that they would refer their patients for physiotherapy. To increase the referral of physiotherapy, 53 (55 %) believed there should be awareness programs among doctors, 60 (64 %) said the awareness should be also among students, while 43 (46 %) believed introducing physiotherapy subject into their course work would help. Only 32 (34 %) were of opinion that elective posting in physiotherapy department would be beneficial.

Respondents had a positive opinion for physiotherapy as a profession with 15 (16 %) mentioning it as excellent, 35 (37%) very good and 39 (41%) good. However, 25 (27%) of them believed physiotherapists are not well paid. Medical Intern's overall average knowledge about modalities used in physiotherapy was 26%. Knowledge about role of physiotherapy practice had mixed responses (Figure 2).

Table 2 shows the demographic characteristics of the medical interns who participated in the study (Table 2).

**Table 2:** Demographic characteristics of the medical interns who participated in the study.

Age	n (%)
22-26	74 (78.7)
27-31	20 (21.27)
Sex	
Male	62 (65.95)
Female	32 (82.05)
Religion	
Hindu	89 (94)
Buddhist	3 (3.2)
Muslim	2 (2.1)
Ethnicity	
Brahmin/Chhetri	51 (54.3)
Newars	9 (9.6)
Janajati	3 (3.2)
Madhesi	21 (22.3)
Dalit	2 (2.1)
Others	8 (8.5)

## DISCUSSION

Physiotherapy is very essential for treatment in acute, sub-acute and chronic phase of treatment. Although physiotherapists are involved in patient management along with medical doctors, the results of our study and other similar studies show that the medical intern's knowledge of physiotherapy is not very good. Most of the interns in our study i.e., 78 (83%) had inadequate knowledge about physiotherapy practice. However, a study done in India found that the knowledge of physiotherapists was 42% and awareness was 45%.^4^ In another study done in Nigeria, it was reported that the medical students had a good knowledge (20.25±4.50 and 18.77±4.60 for males and females respectively) and fair perception (32.70±7.20 and 34.33±7.30 for males and females respectively) of physiotherapy. This may be due to the formal education received about physiotherapy during their classroom lectures. The author of this study had suggested that inter-professional courses and communication should be given greater attention during medical training.^7^ Another study done among medical doctors also concluded by stating that they do not have an adequate knowledge and understanding of physiotherapy profession.^8^ If a physician were well informed about how physiotherapist could help the treatment of patients and prevent the development of complications, hospital stays could be decreased to a larger extent^2,9^, decreasing the burden on the healthcare system. Patients' care yields better outcomes when a comprehensive approach that encompasses all relevant healthcare professionals is used.^10^

From our results above we find 88% medical interns agreed to refer patients to physiotherapy. Similar results were derived from study by Shemjaz, et al.^4^ where 95 % of interns agreed to refer their patients for physiotherapy.

At the end of the study, we can clearly understand that the medical interns did not have adequate awareness and knowledge regarding physiotherapy which may hamper the effective and timely referral of patients. The reason for the lack of awareness and knowledge may be many but it is sure that there exists a need for educating the future medical professionals about the various aspects of physiotherapy so that there can be a betterment of health care system as a whole.

Also, in our study we find that only 24.5% of medical interns know that WHO has classified physiotherapists as independent practitioners. This can also be said about major practicing doctors and senior faculties. 58.5% of respondents said physiotherapy has a definite assessment protocol and only 28% had a knowledge that exercise prescription is also done for any condition in physiotherapy. This is also probably because of the lack of awareness at the level of regulating bodies in the country that we are following the hierarchy where physiotherapists are not independent and have to work under other departments. This has resulted in quality of physiotherapy being compromised as physiotherapists cannot take independent decisions.

Limitations of study were that the data collection was not done using random sampling methods, and it was conducted in single centre.

## CONCLUSIONS

The prevalence of adequate knowledge is less in our study which is similar to other studies done in similar settings. The medical interns are future doctors so educating them about physiotherapy by adopting different strategies is very important for timely and appropriate referral of patient which in turn results in a better patient care and overall benefit of health care system.
